# Diarrhea and associated factors among under five children in sub-Saharan Africa: Evidence from demographic and health surveys of 34 sub-Saharan countries

**DOI:** 10.1371/journal.pone.0257522

**Published:** 2021-09-20

**Authors:** Getu Debalkie Demissie, Yigizie Yeshaw, Wallelign Aleminew, Yonas Akalu

**Affiliations:** 1 Department of Health Education and Behavioral Sciences, Institute of Public Health, College of Medicine and Health Sciences, University of Gondar, Gondar, Ethiopia; 2 Department of Human Physiology, School of Medicine, College of Medicine and Health Sciences, University of Gondar, Gondar, Ethiopia; 3 Department of Epidemiology and Biostatistics, Institute of Public Health, College of Medicine and Health Sciences, University of Gondar, Gondar, Ethiopia; National Research Centre of Egypt, EGYPT

## Abstract

**Introduction:**

Diarrhea is responsible for the death of more than 90% of under-five children in low and lower-middle income countries. Regionally, South Asia and sub-Saharan Africa accounted for 88% of deaths with the same age group. Therefore, the aim of this study was to determine the prevalence and associated factors of diarrhea among children under-five years in sub-Saharan Africa.

**Methods:**

The appended, most recent demographic and health survey datasets of 34 sub-Saharan African countries were used to determine the prevalence and associated factors of diarrhea among under-five children in the region. A total weighted sample of 330,866 under-five children were included in the study. Both bivariable and multivariable multilevel logistic regression were done to determine the associated factors of diarrhea among under five children in sub-Saharan Africa. The Odds Ratio (OR) with a 95% Confidence Interval (CI) was calculated for those potential factors included in the final model.

**Result:**

The overall prevalence of diarrhea in this study was 15.3% (95% CI: 15.1–15.4). Those children of mothers aged 15–24 (AOR = 1.26; 95% CI: 1.23, 1.30) and 25–34 years (AOR = 1.15; 95%CI: 1.12, 1.18), those children of mothers with no education (AOR = 1.69; 95%CI: 1.57–1.82), primary education (AOR = 1.73; 95%CI: 1.61–1.86) and secondary education (AOR = 1.49; 95%CI: 1.38–1.59) had higher odds of having diarrhea. Those children from poorest (AOR = 1.14; 95%CI: 1.10, 1.19), poorer (AOR = 1.12; 95%CI: 1.08–1.17), middle (AOR = 1.06; 95%CI: 1.02, 1.10), and richer (AOR = 1.14; 95%CI: 1.04–1.12) households had higher chance of having diarrhea compared to their counterparts.

**Conclusion:**

This study found that the prevalence of childhood diarrhea morbidity in sub-Saharan Africa was high. Maternal age, wealth index, maternal education, maternal occupation, age of child, time of initiation of breast feeding and time to get water source were significantly associated with diarrhea. Therefore, intervention through health education and health promotion for mothers/caretakers who are poor, less educated, and young should be designed to prevent diarrhea in the region.

## Introduction

According to World Health Organization (WHO), diarrhea is defined as the passage of three or more loose or liquid stools per day because of abnormally high fluid content of stool or an abnormal increase in daily stool frequency [1]. Globally, diarrhea is the second-leading cause of death among under-five children, accounting 9% of all under-five deaths [2]. In developing countries, it has been estimated that 1.8 million people die annually due to diarrheal diseases and more than 80% of them are children aged under five years [3, 4]. Diarrhea is responsible for the death of more than 90% of under-five years of age in low and lower-middle income countries, and regionally, South Asia and sub-Saharan Africa (SSA) accounted for 88% of deaths for the same age group [2].

Children with diarrhea will face many problems including loss of appetite, electrolyte deficit, malnutrition, increased risk of developing other infectious diseases and delayed physical growth and mental development [5, 6]. Because of its negative impact on the physical and cognitive development, diarrhea is also associated with multiple problems; causing for 72.8 million disability and adjusted life years, and it worsens the economic situation of families and the healthcare system [7, 8].

Inadequate quantities and quality of drinking water and lack of sanitation facilities cause the death of millions of the world’s poorest people through diarrheal diseases each year [9, 10]. Furthermore, studies revealed that age of child [11–15], maternal education [11, 12, 16], lack of awareness of mothers/caregivers [17, 18], lower socio-economic status [12], distance and source of drinking water [11, 19, 20], latrine and hand washing facilities [21, 22], breast feeding [21, 23], place of residence [14, 22, 24], disposal of children’s stool [24–26], family size [17, 27], number of under-five children in the household [13, 18], maternal age [19, 20] and maternal employment status [13, 17, 19, 28] as the determinant factors of diarrhea among under-five children.

Despite diarrhea among under-five years of age is a huge problem in sub-Saharan Africa, to the best of our knowledge, there is no study that investigates its prevalence and factors associated with it in the region. Hence, this study was conducted to determine the prevalence of diarrhea and associated factors among children under-five years of age in the region. Conducting regional based studies will help to recognize risk factors of diarrheal diseases that enables the concerned bodies to develop appropriate interventions, which might vary depending on the environmental conditions [29].

## Methods

### Data source

This study used the most recent appended demographic and health surveys (DHS) datasets of 34 sub-Saharan countries which were conducted from 2009 to 2018. The DHS is a nationally representative survey, collected every five years to provide population and health indicators at the regional and national levels. Each country used pre-tested and standard DHS questionnaires to collect data. The questionnaire was conceptualized to the different countries context and the data were collected by trained data collectors. The datasets of each sub Saharan country were obtained at https://dhsprogram.com/data/dataset_admin/index.cfm. Five countries with no data on diarrhea among under five children were excluded from the analysis. In this study, a total weighted sample of 330,866 under-five children were included (Table 1).

**Table 1:** List of sub-Saharan countries and their demographic and health survey’s year.

Country name	Survey year	Weighted sample size
Angola	2015/16	12,669
Burkina Faso	2010	14,001
Burundi	2016/17	12,813
Cameroon	2018	9,942
Congo Democratic Republic	2013/14	16,968
Chad	2015	16,745
Comoros	2012	3099
Congo	2011/12	7,750
Cote d’vore	2011/12	6,811
Ethiopia	2016	10,417
Gabon	2012	4,848
Ghana	2014	5429
Gambia	2013	7,586
Guinea	2018	7202
Kenya	2014	18,679
Liberia	2013	6047
Lesotho	2014	2896
Madagascar	2009	11,976
Mali	2018	9,566
Malawi	2015/16	16,548
Mozambique	2011	5303
Nigeria	2018	30,881
Niger	2012	12,268
Namibia	2013	2,553
Rwanda	2014/15	7,692
Sera lone	2016	8,893
Sao tome and Principe	2009	1,741
Senegal	2011	10,893
Togo	2013	6281
Tanzania	2015/16	9,511
Uganda	2016	14,493
South Africa	2016	3,444
Zambia	2018	9,361
Zimbabwe	2015	6055

### Variables of the study

The dependent variable for this study was diarrhea. Diarrhea is a binary outcome determined by asking the mother “has your child had diarrhea in the last 2 weeks?” Accordingly, these children were said to have diarrhea if they have diarrhea 2 weeks preceding the survey and no otherwise [22].

Both individual and community level variables were considered as independent variables. The individual level variables were age of mothers, education level of mothers, wealth index, employment status of mothers, family size, number of under five children, duration of breast feeding, age of the child, sex of the child, time of initiation of breast feeding, birth order, taking of vitamin A in the last 6 months, time to get water source and media exposure. Residence and SSA region were considered as the community level factors.

#### Operational definitions

Media exposure: media exposure is a composite variable generated by the aggregation of listening radio, reading newspaper and watching television and it was dichotomized as yes “if the mother had exposure to either of the above three mentioned media sources” and no “if she didn’t have exposure to all of the three media sources.

Wealth index: In the DHS, an asset-based approach was used to calculate the wealth index. The collected information was on ownership of a range of durable assets (e.g. car, refrigerator, and television) and housing characteristics (e. g. material of dwelling floor and roof and toilet facilities). Women were asked whether they had the above assets or not. These scores were derived using principal component analysis and ranked into the poorest, poorer, middle, richer, and richest categories. The wealth quintiles are expressed in terms of quintiles of individuals in the population rather than quintiles of individuals at risk for anyone’s health or population indicator [30].

Currently working: Women currently working was to mean women employed in the last 12 months [30]. Residence: The definition of a cluster as urban or rural is made according to the definition used in each country. The traditional distinction between urban and rural areas within a country has been based on the assumption that urban areas, no matter how they are defined, provide a different way of life and usually a higher standard of living than rural areas [30].

Improved source of water: if the source of water is from piped into dwelling, piped to yard/plot, public tap/standpipe, piped to neighbor, tube well or borehole, protected well, protected spring, rainwater and bottled water while the others sources are unimproved [30].

### Data analysis procedure

We used STATA 14 software to extract, recode and analyze the data. The data were weighted before doing any statistical analysis to restore the representativeness of the sample and get a reliable estimate and standard error. Sample weights were calculated to six decimals but are presented in the standard recode files without the decimal point. They need to be divided by 1,000,000 before use to approximate the number of cases. There are four main sampling weights in DHS surveys: household weights, household weights for the men’s subsample, individual weights for women, and individual weights for men: for our study, we used individual weight for women (v005) which is the household weight (hv005) multiplied by the inverse of the individual response rate for women in the stratum. The whole procedure of weighting and its rationale is found on the guide of DHS statistics [30].

Due to the correlated nature of DHS data, measures of community variation/random-effects such as Interclass Correlation Coefficient (ICC), Median Odds Ratio (MOR), and Proportional Change in Variance (PCV) were calculated. According to the values of these measures the use of multilevel logistic regression model is more appropriate than ordinary logistic regression. To choose the best fitted model, first we developed four models and compared them with deviance. The first one is the null-model; a model with no independent variable, the second model is model I; a model that has individual-level factors only, model II; a model with community-level factors only and model III; a model that contain both individual and community level independent variables. Of the four models fitted, model III was selected as the best fitted model (it had the lowest deviance).

Then after, both bivariable and multivariable multilevel logistic regression was conducted to determine the prevalence and associated factors of diarrheal disease in SSA. All variables with a p value < 0.2 at bi-variable analysis were entered into the multivariable logistic regression model [31]. In the final model p value ≤ 0.05 was used to declare statistically significant variables.

### Ethics consideration

We used a secondary analysis of DHS data. Therefore, obtaining ethical approval is not needed. However, we have received a permission letter to download and use the data files from DHS Program.

## Results

### Sociodemographic characteristics of the respondents

The total weighted samples of 330,866 under-five children were included in this study. Of them, 166,758 (50.4%) were males and 66,668(20.2%) of them were in the age group of 12–23 months. Of the respondents, 159,678(48.3%) were in the age group of 25–34 years. More than one-third of their mothers; 127,248 (38.5%) had no education and almost two-third of their families, 212,839 (64.3%) had media exposure (Table 2)

**Table 2:** Sociodemographic characteristics of the respondents in sub-Saharan Africa.

Variable	Category	Weighted Frequency	Percent (%)
Sex of child	Male	166,758	50.4
	Female	164,108	49.6
Age of child (in months)	0–11	70,585	21.3
	12–23	66,668	20.2
	24–35	64,362	19.5
	36–47	65,710	19.9
	48–59	63,539	19.1
Residence	Urban	102,378	30.9
	Rural	228,486	69.1
SSA region	East Africa	105,228	31.8
	West Africa	112,848	34.1
	South Africa	49,244	14.9
	Central Africa	63,543	19.2
Age of mothers(years)	15–24	96,254	29.0
	25–34	159,678	48.3
	35 and above	74,934	22.7
Education level of mothers	No formal education	127,248	38.5
	Primary	114,795	34.7
	Secondary	77,529	23.4
	Higher	11,293	3.4
Working status of mothers	Currently working	213,046	64.4
	Currently	117,820	35.6
	Not working		
Family size	<5	135,319	40.9
	≥5	195,547	59.1
Number of under five children in the house hold	≤2	241,075	72.9
	3–4	74,830	22.6
	≥5	14,961	4.5
Wealth index	Poorest	74,457	22.5
	Poorer	71,896	21.7
	Middle	66,511	20.1
	Richer	62,645	18.9
	Richest	55,354	16.8
Media exposure	Yes	212,839	64.3
	No	118,026	35.7

### Behavioral and environmental characteristics of respondents

More than half of the children; 175,776 (53%) were initiated breast feeding within 1 hour of birth. Of the respondents; 213,442 (64.5%) used improved water source and more than half of the respondents; 185,223 (56%) need ≥30 minutes to reach to water source (Table 3).

**Table 3:** Behavioral and environmental characteristics of respondents in sub-Saharan Africa.

Variable	Category	Weighted Frequency	Percent (%)
Breast feeding duration(in month)	0–23 months	303,329	91.7
	24–59 months	27,536	8.3
Initiation of breast feeding	Within 1 hr.	175,776	53.0
	After 1 hr.	155,089	47.0
Birth order	First	72,659	22.0
	Second	63,909	19.3
	3^rd^ and above	194,298	58.7
Vitamin A given in the last 6 months	Yes	176,089	53.2
	No	154,778	46.8
Water source	Improved	213,442	64.5
	Un improved	117,423	35.5
Time to get water source	<30 minutes	145,467	44.0
	≥30 minutes	185,223	56.0

### Random effect analysis

The random-effects model result showed that there is significant clustering of diarrheal disease among under-five children across the communities (OR of community level variance = 0.03, 95% CI = (0.03–0.04). The value of ICC in the null model revealed that 0.9% of the overall variation of diarrheal disease among under-five children was attributed to cluster variability. The 1.18 MOR value of the null model also indicates the presence of variation in the diarrheal disease among under-five children between clusters. It means if we randomly select children from different clusters, those children at the cluster with higher diarrhea had 1.18 times higher chance of developing diarrhea compared to their counterparts.

As you can see in the Table 4 below, model III has the lowest deviance value. Hence, it was selected as best fitted model (Table 4).

**Table 4:** Model comparison and random effect analysis result.

Parameters	Null model	Model I	Model II	Model III
Community level variance	0.03(0.03–0.04)	0.03(0.02–0.04)	0.03(0.02–0.04)	0.03(0.02–0.03)
ICC	0.9%	0.85%	0.88%	0.83%
MOR	1.18	1.17	1.18	1.17
PCV	Ref	9.67%	6.45%	12.9%
**Model fitness**				
Deviance	286643.58	274480.28	286202.12	274145.4

### Prevalence of diarrhea and associated factors among under five children

The overall prevalence of diarrhea in this study was 15.3% (95% CI: 15.1–15.4). Maternal age, wealth index, maternal education, SSA region, maternal occupation, number of under five children, children age, time of initiation of breast feeding and time to get water source were significantly associated with diarrhea. The odds of diarrhea among children of mothers aged 15–24 and 25–34 years was 1.26 (AOR = 1.26; 95% CI: 1.23, 1.30) and 1.15 (AOR = 1.15; 95% CI: 1.12, 1.18) times higher as compared to mothers aged 35 and above years respectively.

Those children’s of mothers with no education, primary and secondary level of education had about 1.69 (AOR = 1.69; 95% CI: 1.57–1.82), 1.73 (AOR = 1.73; 95% CI: 1.61–1.86) and 1.49 (AOR = 1.49; 95% CI: 1.38–1.59) times higher odds of developing diarrhea compared to children of mothers with higher level of education respectively. The odds of getting diarrhea for the children of working mothers was increased by 12% (AOR = 1.12; 95%CI: 1.10, 1.15) as compared to those children of non-working mothers. The odds of getting diarrhea was increased by 14% (AOR = 1.14; 95%CI: (1.09, 1.19) among households with ≥ 5 under-five children compared to those households with ≤2 under-five children.

The chance of developing diarrhea from poorest, poorer, middle and richer households was increased by 14% (AOR = 1.14; 95%CI: 1.10, 1.19), 12% (AOR = 1.12; 95%CI: 1.08–1.17), 6% (AOR = 1.06; 95%CI: 1.02, 1.10), and 14% (AOR = 1.14; 95%CI: 1.04–1.12) as compared to the richest households respectively. Children with age group of 12–23 months were about 1.5 times (AOR = 1.50; 95%CI: 1.46, 1.54) more likely to develop diarrhea when compared with children with age group of 0–11 months. However, children beyond this age group were less likely to develop diarrhea compared to children in the age group of 0–11 months.

Children born to mothers who were initiated breastfeeding after 1 hour of birth were 1.19 times more likely to manifest with diarrhea (AOR = 1.19, 95% CI: 1.16–1.21) when compared with their counter parts. The odds of developing diarrhea was increased by 3% (AOR = 1.03; 95%CI:1.01,1.05) among children whose mother took 30 and more minutes for getting drinking water when compared with children whose mother took less than 30 minutes (Table 5)

**Table 5:** Multilevel regression analysis of diarrhea among under-five children in sub-Saharan Africa.

	Diarrhea		Odds ratio	
Variables	Yes, No (%)	No, No (%)	COR(95%CI)	AOR(95%CI)
			
Age of mothers (in years)				
15–24	18,056 (18.8)	78,196 (81.2)	1.48(1.44–1.52)	1.26(1.23–1.30)*
25–34	23,885 (15.0)	135,792 (85.0)	1.15(1.12–1.18)	1.10(1.08–1.13)*
35 and above	9,963 (13.3)	64,970 (86.7)	1	1
Residence				
Urban	15,584 (15.2)	86,794 (84.8)	1	1
Rural	36,321 (15.9)	192,165 (84.1)	1.08(1.06–1.10)	0.97(0.94–1.01)
Region				
East Africa	16,922 (16.0)	88,305 (84.0)	1.16(1.12–1.19)	1.15(1.11–1.18)*
West Africa	16,278. (14.4)	96,569 (85.6)	1.04(1.01–1.07)	0.99(0.97–1.03)
South Africa	7,183 (14.6)	42,061 (85.4)	1	1
Central Africa	11,520 (18.1)	52,022 (81.8)	1.31(1.27–1.35)	1.25(1.21–1.29)*
Education level of mothers				
No education	19,799 (15.5)	107,447 (84.5)	1.69(1.58–1.81)	1.69(1.57–1.82)*
Primary	19,305 (16.8)	95,490 (83.2)	1.87(1.74–2.00)	1.73(1.61–1.86)*
Secondary	11,676 (15.0)	65,852 (85.0)	1.61(1.49–1.72)	1.49(1.38–1.59)*
Higher	1,124 (10.0)	10,168 (90.0)	1	1
Mothers’ occupational status				
working	11,632 (16.0)	61,026 (84.0)	1.07(1.05–1.09)	1.12(1.10–1.15)*
not working	9,902 (15.5)	54,007 (84.5)	1	1
Number of under5 children				
≤2	37,643 (15.6)	203,431 (84.4)	1	1
3–4	11,679 (84.4)	11,679 (15.6)	0.99 (.97–1.01)	0.98(0.96–1.01)
≥5	12,377 (82.7)	2,583 (17.3)	1.13 (1.08–1.18)	1.14(1.09–1.19)*
Wealth index				
Poorest	12,384 (16.6)	62,073 (83.4)	1.27(1.23–1.31)	1.14(1.10–1.19)*
Poorer	11,818 (16.4)	60,078 (83.6)	1.24(1.19–1.27)	1.12(1.08–1.17)*
Middle	10,146 (15.3)	56,364 (84.7)	1.15(1.11–1.19)	1.06(1.02–1.10)*
Richer	9,874 (15.8)	52,770 (84.2)	1.14(1.09–1.18)	1.08(1.04–1.12)*
Richest	7,681 (13.9)	47,672 (86.1)	1	1
Age of child (months)				
0–11	13,152 (18.6)	57,432(81.4)	1	1
12–23	16,991 (25.5)	49,677 (74.5)	1.48(1.44–1.52)	1.50(1.46–1.54)*
24–35	10,631 (16.5)	53,730 (83.5)	0.86(0.83–0.88)	0.87(0.85–0.89)*
36–47	6,681 (10.2)	59,028 (89.8)	0.49(0.48–0.51)	0.49(0.48–0.52)*
48–59	4,449 (7.0)	59,090 (93.0)	0.33(0.31–0.34)	0.34(0.32–0.35)*
Breast feeding duration(in month)				
0–23 months	48,097 (15.9)	255,231 (84.1)	1	1
24–59 months	3,808 (13.8)	23,728 (86.2)	0.85(0.82–0.86)	0.97(0.93–1.00)
Initiation of breast feeding				
Within 1 hr.	25,507 (14.5)	150,268 (85.5)	1	1
After 1 hr.	26,398(17.0)	128,691 (83.0)	1.18 (1.16–1.20)	1.19(1.16–1.21)*
Time to get water source				
<30 minutes	22,380 (15.4)	123,085 (84.6)	1	1
≥30 minutes	29,495(15.9)	155,727 (84.1)	1.01(1.02–1.03)	1.03(1.01–1.05)*

*P-value<0.05

## Discussion

The aim of this study was to determine the prevalence of diarrhea and associated factors among under-five children in sub-Sahara. The two-week period prevalence of childhood diarrhea morbidity in this study was 15.3% (95% CI: 15.1–15.4). This finding is higher than a study conducted in Mesoamerica; 13.0% [15], Vietnam;11% [32] and India; 5% [33]. The possible reason might be due to environmental and infrastructural differences such as accessibility of water, presence and usage of latrine, availability of hand washing facilities and ways of waste disposal [34]. Therefore, there is a need to increase the access of these facilities in the region through integrated and comprehensive approach to reduce diarrhea related morbidity and mortality among under-five children [35]. However, this finding is lower than a study conducted in Yemen, 29.1% [36], Afghanistan, 26.2% [11] and India,25.2% [37]. These variations may also be due to difference in knowledge, attitude and practices about diarrheal disease.

This study showed that children to young mothers’ group had higher risk of diarrhea than those of children to older mother’s group. This finding is in line with other studies conducted in low and middle- income countries [19, 20, 38, 39]. The possible reason might be the greater experience of older women in child care and younger mothers might have less understanding and knowledge about diarrheal disease, mode of transmission and pathogens spread in the household compared to the older ones. So that health education intervention should consider young mothers as one target audience to prevent diarrhea among under-five children.

This study revealed that the prevalence of diarrhea was higher among children whose mothers had secondary education and below. This finding is similar to a study conducted in Nepal [40] and Brazil [41]. Women education may enhance knowledge, attitude and practice of basic preventive measures like proper breastfeeding, child feeding, water treatment and, healthier childcare [11, 42]. This indicates the importance of improving the contents and quality of education (e.g. including health education and promotion in the school curriculum including low levels of education).

In agreement of studies conducted in Pakistan and Vietnam [43], the current study revealed that a higher risk of diarrhea among under-five children was found in households with five or more under-five children. The possible reason might be when the number of under-five children in a household increases, the risk of exposure to germs and pathogens increases and parents’ attention and quality of care decreases because of the incapability of the mothers to care for a large number of children. This indicates that child spacing might have positive influence to prevent diarrhea. Hence, it should be considered as one main intervention to reduce morbidity and mortality of under-five children related to diarrhea.

The present study also showed that children to mothers who currently work had higher odds of diarrhea compared to mothers who do not work currently. This finding is in line with other studies in Ethiopia and some sub-Saharan countries [13, 14, 17]. This could be because mothers who currently work might not have enough time to care their children since they spend most of their time at work to increase family income while mothers who do not work currently usually have time to care their children and can minimize the exposure of their children from contaminated objects.

In this study higher risk of diarrhea was observed among under five children from lower wealth index. This finding is consistent with finding in Iraq [44] and India [45]. Wealth has a direct implication on access to sanitation services and basic water. Households in poor conditions are more likely to use poor sanitation sources and unimproved water, thus children in these conditions are highly susceptible to infections including diarrhea [46, 47].

The current study showed that children who were initiated breast feeding within an hour of birth had less chance to develop diarrhea. This finding is similar to other studies in Brazil and Cameroon [18]. This implies that mothers who give birth should be advised that to give breast feeding within an hour for the protective effect of initiation of breastfeeding for infectious diseases in general and diarrhea in particular [48].

This study revealed that age of the child was significantly associated with the development of diarrhea. The risk of developing diarrheal diseases among children aged from 12 to 23 months was high compared with children aged 0 -11months. This finding is similar to studies conducted in Bangladesh and Indonesia [49]. This might be due to children whose age at 12 to 23 months starts crawling and moving around the house thus they can easily ingest dirty or other contaminated objects [25]. This period is also time of stopping breast milk for most of under-five children, so that when breast feeding stops, infants are exposed to food borne germs and loss the protection of breast milk’s anti-infective properties [50].

The present study also showed that children from households who spend greater than 30 minutes and above to get drinking water source were more vulnerable to diarrhea compared with those who spend below 30 minutes. This finding is in agreement with other studies [22, 51]. In fact, water supply sources does not necessarily mean accessing to clean drinking water but the time burden of water fetching might influence the volume of water collected by households as well as time spent on child care. Additionally, the association of a higher risk of diarrhea with public standpipe water can be explained by water contamination during transportation. Hence, improvements of ease of access and quality of water is recommended as preventive measures to control diarrhea [52].

One of the strength of this study is the use of huge and representative datasets of 34 sub-Saharan countries which makes the finding of the study to be generalizable for the region. The other strength of this study is the use of multilevel modeling, a model that accounts the nested/hierarchical nature of the data, to get reliable estimates. However, this study was not free from limitation. The limitation of this study was that we were unable to assess some water and sanitation related variables which could have association with the diarrheal disease among under five children. The wide variation in the year of national survey of the various countries (2009–2018) was also another limitation.

## Conclusion

The prevalence of childhood diarrhea in sub-Saharan Africa was high. Maternal age, wealth index, maternal education, SSA region, maternal occupation, number of under five children, children age, time of initiation of breast feeding and time to get water resource were significantly associated with diarrhea. Therefore, health education and health promotion intervention for mothers/caretakers who are poor, less educated, and young should be designed to prevent diarrhea in the region. Additional interventions on improving water source and family planning also should be designed.

## List of abbreviations

AOR: Adjusted Odds Ratio
DHS: Demographic and health survey
ICC: Interclass Correlation Coefficient
MOR: Median Odds Ratio
PCV: Proportional Change in Variance
SSA: sub-Saharan Africa
WHO: World Health Organization

## References

[1] UNICEF/WHO. Why children are still dying and what can be done? 2009.

[2] UNICEF. One is Too Many: Ending Child Deaths from Pneumonia and Diarrhoea. https://www.unicef.org/publications/files/UNICEF-Pneumonia-Diarrhoea-report-2016-web-version5.pdf. 2019.

[3] Bakir H, Hadi M, Jurdi M. Towards a renewed public health regulatory and surveillance role in water, sanitation and hygiene. Eastern Mediterranean Health Journal 2017;23(8):525–6. 29105042

[4] Hodge J, Chang HH, Boisson S, Collin S, M. Peletz R, Clasen T. Assessing the association between thermotolerant coliforms in drinking water and diarrhea: an analysis of individual-level data from multiple studies. Environmental Health Perspectives 2016;124(10):1560–7. doi: 10.1289/EHP156 27164618 PMC5047765

[5] World Health Organization. Hospital care for children. Geneva: World Health Organization, pp. 109–120. 2005.

[6] UNICEF.State of the world children. UNICEF,. 1998.

[7] Aikins M, Armah G, Akazili J, Hodgson A. Hospital health care cost of diarrheal disease in Northern Ghana. Journal of Infectious Diseases. 2010;202((Suppl_1)): S126–S30. doi: 10.1086/653573 20684692

[8] Gakidou E, Afshin A, Abajobir AA, Abate KH, Abbafati C, Abbas KM, et al. Global, regional, and national comparative risk assessment of 84 behavioural, environmental and occupational, and metabolic risks or clusters of risks, 1990–2016: a systematic analysis for the Global Burden of Disease Study 2016. The Lancet. 2017;390 (10100):1345–422. doi: 10.1016/S0140-6736(17)32366-8 28919119 PMC5614451

[9] Bartlett S. Water, sanitation and urban children: the need to go beyond ‘improved’ provision. Environ Urban 2003;15:5770.

[10] Fewtrell L, John C. Water, sanitation and hygiene interventions and diarrhoea: A systematic review and meta-analysis. Health Nutrition and Population Discussion Paper 2004.

[11] Nasir W, Saw Y1, Jawid S, Kariya T, Yamamoto E, Hamajima Y. Determinants of diarrhea in children under the age of five in Afghanistan: a secondary analysis of the Afghanistan Demographic and Health Survey 2015. Journal of medical sciences. 2020;82:545–56. 10.18999/nagjms.82.3.545 PMC7548244 33132438

[12] Escobar A, Coimbra C, Horta B, Santos R, Cardoso A. Diarrhea and health inequity among Indigenous children in Brazil: results from the First National Survey of Indigenous People’s Health and Nutrition. BMC Public Health. 2015;15:191. doi: 10.1186/s12889-015-1534-7 25880758 PMC4349470

[13] Atnafu A, Sisay M, Demissie G, Tessema Z. Geographical disparities and determinants of childhood diarrheal illness in Ethiopia: further analysis of 2016 Ethiopian Demographic and Health Survey. Tropical medicine and health. 2020;48(64). doi: 10.1186/s41182-020-00252-5 32774127 PMC7397587

[14] Bado A, Susuman A, Nebie E. Trends and risk factors for childhood diarrhea in sub-Saharan countries (1990–2013): assessing the neighborhood inequalities. 2016. 10.3402/gha.v9.30166 PMC4865764 27174860

[15] Danny VC, Bernardo H, Claire RM, Sima SD, Marielle CG, Annie H, et al. Diarrhea Prevalence, Care, and Risk Factors among Poor Children Under 5 Years of Age in Mesoamerica. J Trop Med Hyg 2016;94(3):544–52. 10.4269/ajtmh.15-0750 PMC4775889 26787152

[16] Hajara A, Faisal M, Rabi’ U. Risk factors associated with prevalence of diarrhea among under 5 years children in low socioeconomic area of urban Bangladesh. International Research Journal of Natural and Applied Sciences. 2017;4(12).

[17] Agegnehu M, Zeleke L, Goshu Y, Ortibo Y, Adinew Y. Diarrhea prevention practice and associated factors among caregivers of under-five children in Enemay District, Northwest Ethiopia. Hindawi: Journal of Environmental and Public Health. 2019. doi: 10.1155/2019/5490716 31214265 PMC6535883

[18] Tambe A, Nzefa L, Nicoline N. Childhood diarrhea determinants in sub-Saharan Africa: a cross-sectional study of Tiko-Cameroon. Challenges 2016;6:229–43.

[19] Hussien H. Prevalence of Diarrhea and Associated Risk Factors in Children Under Five Years of Age in Northern Nigeria: A Secondary Data Analysis of Nigeria Demographic and Health Survey 2013. 2017.

[20] Moon J, Choi J, Oh J, Kim K. Risk factors of diarrhea of children under five in Malawi: based on Malawi Demographic and Health Survey 2015–2016. Journal of Global health science. 2019;1(2):45.

[21] Dagnew A, Tewabe T, Miskir Y, Eshetu T, Kefelegn W, Zerihun K, et al. Prevalence of diarrhea and associated factors among under-five children in Bahir Dar city, Northwest Ethiopia: a cross-sectional study. 19. 2019;BMC infectious Diseases(417). 10.1186/s12879-019-4030-3 PMC6518740 31088387

[22] Gedamu G, Kumie A, Haftu D. Magnitude and Associated Factors of Diarrhea among Under Five Children in Farta Wereda, North West Ethiopia. 25. 2017;4:199–207.

[23] Fetensa G, Fekadu G, Tekle F, Markos G, Etafa W, Hasen H. Diarrhea and associated factors among under-5 children in Ethiopia: A secondary data analysis. SAGE open medicine. 2020;8:1–8. doi: 10.1177/2050312120944201 32821387 PMC7406926

[24] Mengistie B, Berhane Y, Worku A. Prevalence of diarrhea and associated risk factors among children under-five years of age in Eastern Ethiopia: a crosssectional study. Open J Prev Med. 2013;3(07):446.

[25] Desalegn M, Kumie A, Tefera W. Predictors of under-five childhood diarrhea: Mecha District, West Gojjam, Ethiopia. Ethiopian Journal of Health Development. 2011;25:174–232.

[26] Mediratta PR, Feleke A, Moulton HL, Yifru S, Sack BR. Risk factors and case management of acute diarrhoea in North Gondar zone, Ethiopia. Journal of Health, Population and Nutrition. 28. 2010:253–63. doi: 10.3329/jhpn.v28i3.5552 20635636 PMC2980890

[27] Kunii O, Nakamura S, Abdur R, Wakai S. The impact on health and risk factors of the diarrhoea epidemics in the 1998 Bangladesh floods. Public Health. 2002;116:6874. doi: 10.1038/sj.ph.1900828 11961673

[28] Hatt L, Waters H. Determinants of child morbidity in Latin America: a pooled analysis of interactions between parental education and economic status. Soc Sci Med. 2006;62:37586. doi: 10.1016/j.socscimed.2005.06.007 16040175

[29] Kumar SG, Subita L. Diarrhoeal diseases in developing countries: a situational analysis. Kathmandu University Medical Journal 2012;10(2):83–8. doi: 10.3126/kumj.v10i2.7351 23132483

[30] Croft T, Marshall A, Allen C, Arnold F, Assaf S, Balian S. Guide to DHS statistics. Rockville: ICF. 2018.

[31] Maldonado G, Greenland S. Simulation study of confounder-selection strategies. American Journal of Epidemiology. 1993;138: 923–36. doi: 10.1093/oxfordjournals.aje.a116813 8256780

[32] GH. A. Determinants of early childhood morbidity and proper treatment responses in Vietnam: results from the Multiple Indicator Cluster Surveys, 2000–2011. Glob Health Action 2016. doi: 10.3402/gha.v9.29304 26950559 PMC4780114

[33] Gupta A, Et a. Study of the prevalence of diarrhea in children under the age of five years and its association with wasting. Indian J Sci Res 2014;7(1):1315–18.

[34] Alebel A, Tesema C, Temesgen B, et a. Prevalence and determinants of diarrhea among under-five children in Ethiopia: a systematic review and meta-analysis. PLoS ONE. 2018;13(6). doi: 10.1371/journal.pone.0199684 29953555 PMC6023116

[35] Katharina D, Patrik T, Jochen R, Michael M. Diarrhoea prevalence in children under five years of age in rural Burundi: an assessment of social and behavioural factors at the household level. Global Health Action,. 2014;7(1). 10.3402/gha.v7.24895 PMC4141944 25150028

[36] Mohanna M, Al-Sonboli N. Prevalence ofdiarrhoea and related risk factors among children aged under 5 years in Sana’a, Yemen. Hamdan Med J. 2018;11:29–33.

[37] Ahmed S, et a. Prevalence of Diarrhoeal disease, its seasonal and age variation in under-fives in. Int J Health Sci. 2010;2(2). PMC3068726 21475494

[38] Finlay J, Ozaltin E, Canning D. The association of maternal age with infant mortality, child anthropometric failure, diarrhoea and anaemia for first births: evidence from 55 low- and middle-income countries. BMJ Open. 2011;1:e000226. doi: 10.1136/bmjopen-2011-000226 22021886 PMC3191600

[39] Danquah L. Risk Factors Associated with Diarrhea Morbidity Among Children Younger than Five Years in the Atwima Nwabiagya District, Ghana: A CrossSectional Study. Sci J Public Health. 2015;3(3):344.

[40] Acharya D, Singh J, Adhikari M, Gautam S, Pandey P, Dayal V. Association of water handling and child feeding practice with childhood diarrhoea in rural community of Southern Nepal. J Infect Public Health. 2018;11(1):69–74. doi: 10.1016/j.jiph.2017.04.007 28576344

[41] Ferrer S, Strina A, Jesus S, et a. A hierarchical model for studying risk factors for childhood diarrhoea: a case–control study in a middle-income country. Int J Epidemiol. 2008;37(4):805–15. doi: 10.1093/ije/dyn093 18515864

[42] Dhingra D, Dabas A, Anand T, Pinnamaneni R. Maternal knowledge, attitude and practices during childhood diarrhoea. Trop Doct. 2018;48:298–300. doi: 10.1177/0049475518787425 30012081

[43] Bui Viet Hung The most common causes and risk factors for diarrhea among children less than five years of age admitted to Dong Anh Hospital, Hanoi, Northern Vietnam. 2006.

[44] Siziya S, Muula A, Rudatsikira E. Diarrhoea and acute respiratory infections prevalence and risk factors among under-five children in Iraq in 2000. Indian J Pediatr. 2009;35(8). doi: 10.1186/1824-7288-35-8 19490665 PMC2687547

[45] Rahman A. Assessing income-wise household environmental conditions and disease profile in urban areas: study of an Indian city. Geo J 2006;65:211–27.

[46] Samwel M, Eddison M, Faith N, Richard S, Elizabeth K, Douglas N. Determinants of diarrhea among young children under the age of five in kenya, evidence from kdhs 2008–09. Etude la Popul Africaine. 2014;28(2):1046–56.

[47] Azage M, Kumie A, Worku A, Bagtzoglou A, Anagnostou E. Effect of climatic variability on childhood diarrhea and its high risk periods in northwestern parts of Ethiopia. PLoS One. 2017;12(10). doi: 10.1371/journal.pone.0186933 29073259 PMC5658103

[48] Lamberti L, Fischer W, Noiman A, Victora C, Black R. Breastfeeding and the risk for diarrhea morbidity and mortality. BMC Public Health 2011;3(15). doi: 10.1186/1471-2458-11-S3-S15 21501432 PMC3231888

[49] Rohmawati N. Factors associated with diarrhea among under five years old children in banten province Indonesia. A secondary analysis of Indonesia maternal socio economic survey 2007 and basic health research: PhD dissertation. 2007.

[50] Alambo K. The Prevalence of Diarrheal Disease in under Five Children and associated Risk Factors in Wolitta Soddo Town, Southern, Ethiopia. ABC research Alert. 2015;3.

[51] Amy J, Pickering, Jennifer D. Freshwater availability and water fetching distance affect child health in Sub-Saharan Africa. Environ Sci Technol. 2012;46:2391–7. doi: 10.1021/es203177v 22242546

[52] Cairncross S, Hunt C, Boisson S, Bostoen K, Curtis V, Fung ICH, et al. Water, sanitation and hygiene for the prevention of diarrhoea. Int J Epidemiol. 2010;39(Suppl. 1): i193–i205. doi: 10.1093/ije/dyq035 20348121 PMC2845874

